# Common environmental chemicals do not explain atopy contrast in the Finnish and Russian Karelia

**DOI:** 10.1186/s13601-016-0103-1

**Published:** 2016-04-04

**Authors:** Jyri-Pekka Koskinen, Hannu Kiviranta, Erkki Vartiainen, Pekka Jousilahti, Tiina Vlasoff, Leena von Hertzen, Mika Mäkelä, Tiina Laatikainen, Tari Haahtela

**Affiliations:** Department of Allergy, Skin and Allergy Hospital, Helsinki University Hospital, Jatasalmentie 14a, 00830 Helsinki, Finland; Department of Health Protection, National Institute for Health and Welfare, Kuopio, Finland; Department of Health, National Institute for Health and Welfare, Helsinki, Finland; North Karelia Center for Public Health, Joensuu, Finland; Institute of Public Health and Clinical Nutrition, University of Eastern Finland, Kuopio, Finland; Hospital District of North Karelia, Joensuu, Finland

**Keywords:** Allergy, Atopy, Environmental chemicals, Finnish Karelia, Russian Karelia

## Abstract

**Background:**

Atopic allergy is much more common in Finnish compared with Russian Karelia, although these areas are geographically and genetically close. To explore the role of environmental chemicals on the atopy difference a random sample of 200 individuals, 25 atopic and 25 non-atopic school-aged children and their mothers, were studied. Atopy was defined as having at least one positive skin prick test response to 14 common inhalant and food allergens tested. Concentrations of 11 common environmental pollutants were measured in blood samples.

**Results:**

Overall, the chemical levels were much higher in Russia than in Finland, except for 2,2′,4,4′-tetra-bromodiphenyl ether (BDE47). In Finland but not in Russia, the atopic children had higher concentrations of polychlorinated biphenyls and 1,1-Dichloro-2,2-bis-(p-chlorophenyl)-ethylene (DDE) than the non-atopic children. In Russia but not in Finland, the atopic mothers had higher DDE concentrations than the non-atopic mothers.

**Conclusions:**

Higher concentrations of common environmental chemicals were measured in Russian compared with Finnish Karelian children and mothers. The chemicals did not explain the higher prevalence of atopy on the Finnish side.

## Background

There is a significant difference in the prevalence of atopic conditions between Finnish and Russian Karelia although these two areas are geographically adjacent and inhabitants of these two areas share partly the same ancestry. Living conditions in Russian Karelia are rural and similar to those in Finland in the 1950 s, and the socio-economic gap between the two areas is prominent [1, 2]. Allergic conditions and sensitization to common allergens are much more common both in the Finnish children and adults compared with the Russian Karelia, and the contrast has grown in the younger birth cohorts [3]. Atopic conditions have been linked to changes in lifestyle and environment [4].

Many environmental chemicals and pollutants affect immune function [5]. However, studies on their role in atopic sensitization are scarce [6]. We explored whether the occurrence of atopic conditions in the two areas with high and low atopy prevalence is associated with exposure to environmental chemicals.

## Methods

The study areas and sampling methods have been described in detail earlier [1]. In brief, the study was carried out in North Karelia in eastern Finland and about 180 km away in Pitkäranta Region in the Republic of Karelia in northwestern Russia. Atopy data were obtained by using a self-administered questionnaire and skin prick tests (SPT). SPT was performed using a standard set of nine common inhalant and five food allergens. The subject was considered atopic, if he or she had at least one SPT response with a wheal diameter of 3 mm or larger and the positive and negative controls gave expected results [7]. Originally, 546 child–mother pairs from Finland and 550 from Russia were enrolled in the study. The data for the present analysis was obtained by taking a random subsample of 200 subjects: 25 atopic (SPT positive) and 25 non-atopic (SPT negative) children and their mothers in Finland and Russia. Out of the mothers 20 were atopic in Finland and 23 in Russia. The children were aged 6–15 years (mean 11.2) and mothers 27 to 50 years (mean 37.5). Data on β-HCH were available only for 30 children and their mothers in Finland and Russia and of them 15 children and 11 mothers were atopic in Finland and 20 children and 13 mothers were atopic in Russia.

A total of six polychlorinated biphenyls (PCBs congener numbers 118, 138, 153, 156, 170 and 180), 1,1-Bis-(4-Chlorophenyl)-2,2,2-trichloroethane (DDT),

1,1-Dichloro-2,2-bis-(p-chlorophenyl)-ethylene (DDE), hexachlorobenzene (HCB), β-hexachlorocyclohexane (β-HCH), and 2,2′,4,4′-tetra-bromodiphenyl ether (BDE47) were measured in 200 µl aliquots of sera from children and mothers. Details of sample pretreatment for the persistent organic pollutants have been published recently [8]. In each batch of samples (n = 25), two blanks were included to control for possible laboratory background or cross sample contamination. Additionally, two control serum samples were added to assess the between-assay coefficient of variation which varied between 2.1 and 4.0 %. Average recoveries of measured POPs in control samples were 97–106 % of the certified values. The limits of quantitation (LOQs) for the PCBs, DDE, HCB, β-HCH, and BDE47 were between 2 and 5 pg/mL. The LOQ for DDT was 20 pg/mL. Values below LOQ were measured in the Finnish children as follows N (%): PCB118 6 (12 %), PCB156 22 (44 %), PCB170 4 (8 %), DDT 50 (100 %) and in the Finnish mothers HCH 8 (27 %), DDT 50 (100 %), BDE47 37 (74 %) and PCB156 3 (6 %). The respective values for Russian children were: DDT 17 (34 %) and BDE47 48 (96 %) and for the Russian mothers DDT 16 (32 %) and BDE47 47 (94 %).

In the results we used the raw data of analyses employing the best estimate of the concentration of an analyte, even below the LOQ, in order to avoid extensive amount of LOQs in statistical estimations. The analyses were performed at the National Institute for Health and Welfare, Chemicals and Health Unit, Kuopio, Finland. The Unit is an accredited testing laboratory (T077: ISO/IEC 17025) by Finnish Accreditation Services (FINAS).

Chemical concentrations are reported as unadjusted means with confidence intervals. The distributions of the measured chemicals were skewed so the levels of the POPs were log2-transformed. Correlations between log2-transformed concentrations were tested with Pearson correlations. Mann–Whitney U-test and logistic regression were used for the statistical analyses and p value below 0.05 was considered significant. SPSS version 22 was used for the statistical analyses.

The study protocol was approved by the Coordinating Ethics Committee of the Helsinki University Hospital District. All participants gave their informed consent.

## Findings

**Fig. 1:**
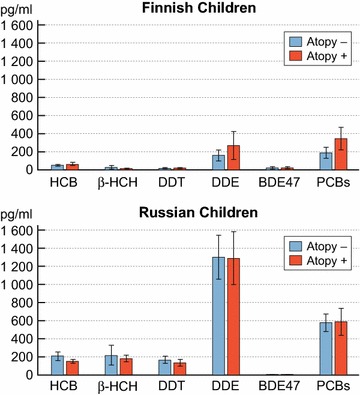
Mean blood concentrations (95 % CI) of 11 chemicals (6 PCBs together) in atopic and non-atopic Finnish children (n = 50, β-HCH n = 30) as well as atopic and non-atopic Russian children (n = 50, β-HCH n = 30)

Altogether, all the chemical concentrations were significantly higher both in the Russian children and mothers (both in SPT positive and -negative subjects) as compared with the Finnish children and mothers (Table 1; Fig. 1). The only exception was BDE47, for which the Finnish children and mothers had higher concentrations than their Russian counterparts, but on both sides the concentrations were very low.

**Table 1:** Mean blood concentrations pg/mL (95 % CI) of 11 chemicals (6 PCBs together) in 200 Finnish and Russian children and mothers

	FIN children	RUS children	FIN mothers	RUS mothers
HCB	52.4 (42.4–62.3)	177.2 (150.6–203.7)	72.1 (61.6–82.6)	275.1 (225.3–324.9)
β-HCH	16.7 (6.65–26.7)	191.6 (151.4–231.9)	23.4 (18.6–28.3)	482.2 (329.2–635.3)
DDT	15.1 (10.8–19.5)	149.1 (121.2–176.9)	20.1 (15.3–24.9)	180.5 (145.5–215.4)
DDE	211.5 (130.8–292.2)	1294.6 (1111.3–1477.9)	424.3 (273.1–575.5)	2593.2 (2003.5–3182.9)
BDE47	19.0 (10.9–27.2)	3.5 (2.5–4.5)	17.8 (4.14–31.4)	4.1 (2.7–5.4)
PCBs	264.2 (194.6–333.8)	582.7 (495.5–669.9)	593.5 (501.5–685.6)	1241.6 (1037.8–1445.3)

All concentrations were significantly higher in the Russian subjects, with the exception of BDE47, the concentration of which was significantly higher in the Finnish subjects. All p values from Mann–Whitney test, comparing Finnish and Russian children and mothers, were <0.01

The Finnish SPT positive children had significantly higher mean concentrations of PCBs and DDE than the SPT negative children. The only difference in the Finnish mothers was that SPT negative mothers had significantly higher mean concentrations of DDE than SPT positive mothers.

On the Russian side, a few significant differences were found between SPT positive and –negative subjects. In children, the SPT negative subjects had higher concentrations of HCB than the SPT positive subjects. In mothers, the SPT positive subjects had higher concentrations of DDE than SPT negative subjects.

We used logistic regression to evaluate the association between chemical concentrations and atopy. SPT status was used as a dependent variable and the log2-transformed chemical concentrations (PCBs, DDT, DDE, β-HCH, HCB, BDE47) as explanatory variables. In the Finnish children DDE (OR 1.77; 95 % CI 1.00–3.11) and PCBs (OR 3.28; 95 % CI 1.40–7.70) were associated with increased risk of atopy. In the Russian children HCB was associated with decreased risk of atopy (OR 0.36; 95 % CI 0.14–0.96). In the Finnish mothers DDE was associated with decreased risk of atopy (OR 0.53; 95 % CI 0.29–0.99) but with increased risk in the Russian mothers (OR 2.03; 95 % CI 1.02–4.02).

## Discussion

The main outcome was that chemical exposure did not seem to explain the atopic disposition (SPT positivity), or it’s differences, in the present Finnish and Russian population samples.

Generally, the chemical levels were higher in the Russian subjects, although atopy is markedly more common on the Finnish side. The statistical differences between atopic and non-atopic individuals did not indicate a consistent pattern. Furthermore, the results from the chemical specific logistic regression analyses indicated some significant but contradictory associations. The lack of consistency might result from relatively small numbers and other confounding factors, but we find it unlikely that the measured chemicals are important reasons for the atopy differences.

It seemed that the Russian population has been exposed more to pesticides and PCBs whereas the Finnish population more to brominated flame retardants, such as BDE47. The usage of pesticides and PCBs in Russia has, according to the present results, been more recent and extensive compared with Finland. Evidence of a more recent use of DDT are the higher concentrations of DDT in the Russian serums. Other explanations for higher concentrations of pesticides in Russia are contaminated soils and poorly maintained stocks as possible sources of these obsolete pesticides.

The higher concentrations of BDE47 in Finland are probably due to more extensive utilization of flame retardants in various electronic devices and furniture together with more time spend indoors with TV, computers, and other electronics.

It has been hypothesized that persistent organic pollutants (POPs) could have an effect on the developing immune system, and biomarkers have been identified in allergic children that indicate exposure to POPs [9, 10]. However previous studies on environmental chemicals and atopy have shown inconsistent results. A study from Denmark demonstrated that prenatal exposure to POPs was positively associated with offspring airway obstruction, but not with atopic sensitization at 20 years of age [11]. Another study from Ukraine and Greenland found only limited evidence that prenatal exposure to POPs would affect childhood asthma and eczema [12]. There is some evidence that exposure to POPs could favour the atopic disposition [6] but also evidence that exposure to PCBs and dioxins could be associated with greater amount of childhood infections and that could even prevent children from developing allergic diseases [13]. Interestingly a recent study from Spain indicated that prenatal exposure to bisphenol A and phthalates, which are widely used in household chemicals might increase the risk asthma symptoms in childhood [14]. Methodological variability and sample sizes may explain some of the inconsistence in the previous studies. It is still uncertain what role POPs could play in the complex process of developing allergic predisposition.

In the present study, the atopic Finnish children showed higher concentrations of PCBs and DDE than the non-atopic children. This could have happened by chance, but atopic individuals often have increased skin and mucous barrier permeability, which also could have an effect. This hypothesis needs to be tested in a larger population sample.

Our results do not indicate that exposure to environmental chemicals would explain the observed difference in atopy prevalence between the Finnish and Russian Karelia.

## Abbreviations

Polychlorinated biphenyls (PCBs): synthetic mixtures of individual chlorinated compounds. They have been widely used for example in electronic industry

1,1-Bis-(4-Chlorophenyl)-2,2,2-trichloroethane (DDT) and 1,1-Dichloro-2,2-bis-(p-chlorophenyl)-ethylene (DDE): DDT is used in agriculture as pesticide and as insecticide to control malaria and typhus. DDE is the main metabolite of DDT in humans. DDT found in serum can be a marker for recent exposure and DDE for older exposure to environmental chemicals

Hexachlorobenzene (HCB): used in agriculture as a fungicide and in the manufacturing process of fireworks, ammunition and synthetic rubber

β-hexachlorocyclohexane (β-HCH): used as a pesticide widely in 1960s and 1970s. Still produced as an unintentional by-product in the chemical industry

2,2′,4,4′-tetra-bromodiphenyl ether (BDE47): added into plastic consumer products because of the flame-retardant feature. Can leach easily into the environment
